# RNA Nanotechnology to Solubilize Hydrophobic Antitumor Drug for Targeted Delivery

**DOI:** 10.1002/advs.201900951

**Published:** 2019-09-30

**Authors:** Xijun Piao, Hongran Yin, Sijin Guo, Hongzhi Wang, Peixuan Guo

**Affiliations:** ^1^ Center for RNA Nanobiotechnology and Nanomedicine The Ohio State University Columbus OH 43210 USA; ^2^ College of Pharmacy Division of Pharmaceutics and Pharmacology The Ohio State University Columbus OH 43210 USA; ^3^ College of Medicine Dorothy M. Davis Heart and Lung Research Institute The Ohio State University Columbus OH 43210 USA; ^4^ James Comprehensive Cancer Center The Ohio State University Columbus OH 43210 USA

**Keywords:** RNA nanotechnology, RNA‐drug conjugation, solubility, targeted delivery

## Abstract

Small‐molecule drugs are used extensively in clinics for cancer treatment; however, many antitumor chemical drugs dissolve poorly in aqueous solution. Their poor solubility and nonselective delivery in vivo often cause severe side effects. Here, the application of RNA nanotechnology to enhance the solubility of hydrophobic drugs, using camptothecin (CPT) for proof‐of‐concept in targeted delivery for cancer treatment is reported. Multiple CPT prodrug molecules are conjugated to RNA oligos via a click reaction, and the resulting CPT‐RNA conjugates efficiently self‐assemble into thermodynamically stable RNA three‐way junction (3WJ) nanoparticles. The RNA 3WJ is covalently linked with seven hydrophobic CPT prodrug molecules through cleavable ester bonds and a folic acid ligand for specific tumor targeting while remaining soluble in aqueous solutions without detectable aggregation at therapeutic dose. This CPT‐RNA nanoparticle exhibits efficient and specific cell binding and internalization, leading to cell apoptosis. Tumor growth is effectively inhibited by CPT‐RNA nanoparticles; the targeted delivery, strengthened by tumor ligand, further enhances tumor suppression. Compared with the traditional formulation, solubilization of CPT in aqueous buffer using RNA nanoparticles as a carrier is found to be safe and efficacious, demonstrating that RNA nanoparticles are a promising platform for the solubilization and the delivery of hydrophobic antitumor drugs.

## Graphical abstract

Robust RNA nanoparticles with a defined three‐way junction structure are used to solubilize a hydrophobic antitumor drug in aqueous solution and improve the safety profile. Targeted delivery of the therapeutic RNA‐drug nanoparticles further increases the tumor suppression efficacy in human tumor xenograft models. This work demonstrates the feasibility of RNA nanotechnology for the solubilization and the delivery of hydrophobic chemotherapeutic drugs.

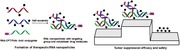

Cancer is caused by the uncontrolled growth and division of cells and is one of the most fatal diseases worldwide.^1^ Small‐molecule antitumor drugs, that either inhibit DNA replication or interfere with microtubule formation, have long been used in cancer treatment.^2^ Despite their sizeable market share, two major issues are often associated with small‐molecule antitumor drugs.^3^ First, some of them are very hydrophobic molecules and dissolve poorly in aqueous solutions. Particulate drug matters in the intravenous (IV) injection can be very harmful to patients due to embolization. As a result, organic solvent, polyethylene glycol (PEG), surfactant, and oil are frequently used as co‐solubilizers to formulate these hydrophobic drugs for clinical IV administration. For example, Cremophor EL and ethanol are used to formulate Paclitaxel.^4^ Similarly, hydrogenated vegetable oil and glyceryl monooleate are used to formulate topotecan,^5^ a camptothecin (CPT) derivative in the market; however, these co‐solubilizers at high concentrations result in adverse side effects such as immune responses, allergies, and hypersensitivity.^6^ The other problem with small‐molecule antitumor drugs is nonspecific delivery into both tumor and healthy tissues. Due to the nonselective cell entry mechanisms of chemical antitumor drugs, both tumor cells and healthy cells are killed, leading to severe side effects and dramatically lowering the patients' life quality. To overcome these two well‐recognized problems, tremendous research effort has been made to design various nanocarriers for the targeted delivery of poorly soluble antitumor drugs. Drugs can be noncovalently encapsulated in liposomes^7^ and polymers^8^ via hydrophobic interaction or covalently conjugated to inorganic nanoparticles^9^ through releasable linkers. Although these colloidal nanocarriers showed promising results in delivery of hydrophobic antitumor drugs, the safety of insoluble nanoparticles is still a concern.^10^ To this end, a completely water‐soluble nano‐vehicle with high loading of hydrophobic antitumor drugs would be ideal for targeted delivery.

Water‐soluble RNA nanoparticles have recently been developed for the efficient delivery of therapeutics.^11^ Among these efforts, our lab focused on a novel delivery system based on robust small‐size RNA nanoparticles (5–40 nm). The core RNA three‐way junction (RNA 3WJ) is derived from naturally occurring packaging RNA of phi29 bacteriophage motor and can be assembled from three short separate RNA oligos (16–20 nt) with high efficiency.[qv: 11a,12] Incorporating 2'‐fluoro (2'‐F) pyrimidines into the RNA nanoparticles dramatically enhances the thermal, chemical, and enzymatic stability,^13^ enabling various in vivo applications. The convenience of RNA nanotechnology is that different functional modules can be readily harbored onto the same RNA nanoparticle by simply mixing different RNA oligos with the desired functionality at equimolar concentration. For example, one therapeutic antisense oligonucleotide, a cancer‐targeting aptamer, and a fluorophore can be displayed on the same RNA nanoparticle for targeted delivery of therapeutics and in vivo tracking at the same time.[qv: 11c,14] RNA nanoparticles have achieved successful in vivo tumor suppression using gene therapeutics such as siRNAs, miRNAs, and anti‐miRNAs.[qv: 11c,15] However, delivery of small‐molecule drugs by RNA nanoparticles remains challenging, due to the limited drug loading capacity. To achieve an effective therapeutic outcome, a large number of small‐molecule drugs are needed to load onto each RNA nanoparticle, making RNA nanoparticle construction additionally complicated.^16^

We herein report the use of water‐soluble RNA 3WJ nanoparticles to solubilize hydrophobic CPT for targeted delivery in human tumor xenograft models. CPT is a potent small‐molecule antitumor drug,^17^ with extremely poor water solubility and instability which impede its clinical use.^18^ Though both small‐molecule prodrug designs^19^ and nanoparticle‐based formulations^20^ have been extensively studied for the delivery of CPT, a water‐soluble RNA nanoparticle formulation with high CPT loading has not been reported. The hydrophilic nature of RNA dramatically increased the aqueous solubility of CPT, eliminating the need of organic solvent, PEG, surfactant, or oil as co‐solubilizers. Our design covalently loaded seven CPT prodrug molecules onto a 54‐nt RNA 3WJ to achieve enhanced tumor suppression with reduced side effects. To increase the internalization of RNA nanoparticles into tumor cells, folic acid (FA) as a targeting ligand was displayed on the CPT‐RNA nanoparticles to specifically recognize folate receptor that was overexpressed on tumor cell surface. Taken together, these features enabled CPT‐RNA nanoparticles enhanced tumor suppression on human tumor xenograft model. This study thus demonstrated the feasibility of using RNA nanoparticles for efficient and safe in vivo delivery of small‐molecule chemotherapeutic drugs.

To conjugate CPT to RNA oligos, we acylated the 20(S)‐hydroxyl group with 6‐azidohexonic acid to form a CPT prodrug. The ester linkage is used for enzymatic release of parent CPT and the azido group is for subsequent conjugation to RNA oligos with alkyne groups via copper(I)‐catalyzed alkyne‐azide cycloaddition. To maximize the therapeutic effect, multiple CPT prodrug molecules were conjugated to one RNA oligo (**Figure** **1**a). The highly efficient click reaction ensures quantitative conjugation of CPT prodrug molecules to the alkyne‐displaying RNA oligos (3WJ_a_‐3alkyne and 3WJ_b_‐4alkyne). CPT conjugation resulted in increased hydrophobicity of CPT‐RNA conjugate compared with RNA as evidenced by the extended retention time on reverse phase high‐performance liquid chromatography (HPLC; Figure 1b and Figure S1, Supporting Information). The conjugation progress can also be conveniently monitored by a denaturing polyacrylamide gel electrophoresis (PAGE). Due to the increased molecular size and hydrophobicity, RNA with more CPT conjugations showed slower migration and the fully conjugated RNA moved the slowest (Figure 1c and Figure S2, Supporting Information). Mass spectrometry also confirmed molecular weight change by three CPT prodrug molecules after the three alkyne groups on RNA were reacted (from 3WJ_a_‐3alkyne to 3WJ_a_‐3CPT; Figure S3, Supporting Information).

**Figure 1:**
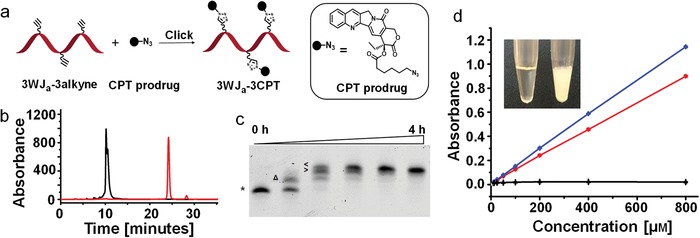
Conjugation of CPT prodrug to RNA (3WJ_a_‐3CPT) and aqueous solubility improvement. a) Illustration of CPT‐RNA conjugation. b) Comparison of reverse‐phase HPLC spectra (absorbance at 260 nm) of 3WJ_a_‐3alkyne (black) and 3WJ_a_‐3CPT after purification (red). c) CPT‐RNA conjugation with increasing reaction time up to 4 h, evaluated by denaturing PAGE (* indicates 3WJ_a_‐3alkyne; ^Δ^ indicates 3WJ_a_‐1CPT; ^>^ indicates 3WJ_a_‐2CPT; ^<^ indicates 3WJ_a_‐3CPT). d) Water solubility comparison (absorbance at 354 nm) of CPT in water (black), CPT‐RNA conjugate in water (red), and CPT in DMSO (blue). Inset photo shows the visual solubility of CPT‐RNA conjugate (left, 8 × 10^−3^ m CPT) and 8 × 10^−3^ m free CPT (right) in water.

Poor solubility of CPT in aqueous solutions has hindered its clinical use in cancer treatment. Although two CPT derivatives are commercially available in the market, a lot of research effort is focused on improving its aqueous solubility by conjugating CPT to hydrophilic moieties including peptides,^21^ dendrimers,^22^ and polymers.[qv: 20a,c,d] Our design of CPT‐RNA conjugates takes advantage of the highly soluble nature of RNA, and results in a dramatic improvement of the aqueous solubility of CPT, as measured by UV/vis spectrometry. Specifically, absorbance at 354 nm, the reference wavelength of CPT, increased proportionally to the concentration of CPT‐RNA conjugate (3WJ_a_‐3CPT) in water, indicating that CPT‐RNA conjugate was completely dissolved (Figure 1d). In contrast, the absorbance of CPT free drug in water after removing the undissolved solid showed no change with the increasing amount of CPT mixed in water, suggesting that solubility of CPT in water is extremely poor (Figure 1d). To confirm the results, dimethyl sulfide (DMSO), a common solvent for CPT, was chosen to dissolve CPT and a similarly proportional increase in absorbance as a function of the same serial concentrations was observed. This suggests the comparable solubilities of CPT‐RNA conjugate in water and free CPT in DMSO. The CPT concentration was only measured up to 800 × 10^−6^ m by UV/vis spectrometry, which was far below the maximum solubility of CPT‐RNA conjugate in water, as a tenfold‐concentrated CPT‐RNA conjugate solution (8 × 10^−3^ m CPT) still showed no visible precipitate (Figure 1d, inset picture). Collectively, the water solubility of CPT was increased by at least 1000‐fold after conjugation to RNA, compared to the reported 2.7 µg mL^−1^ (7.75 × 10^−6^ m) of free CPT in water.^23^

In addition to the improvement of aqueous solubility by CPT‐RNA conjugate, the drug loading capacity was further increased by assembling three single‐stranded RNA conjugates into RNA 3WJ nanoparticles (**Figure** **2**a). In this case, CPT and FA are covalently conjugated to each individual oligo through different chemical reactions; while different oligos are hybridized noncovalently and formed a stable 3WJ via base‐pairing and base‐stacking. Gratifyingly, the assembly efficiency of the core RNA 3WJ was not affected by enhanced loading of CPT. A clear stepwise assembly was observed when adding different RNA oligos on a native PAGE (Figure 2b). The resulting FA‐7CPT‐3WJ nanoparticles, with one FA and seven CPT prodrug molecules, showed the slowest migration in gel (Figure 2b). This assembled RNA nanoparticle displayed a high melting temperature (*T*_m_) of 63.5 °C in physiological phosphate‐buffered saline (PBS) buffer (Figure 2c), as evaluated by temperature‐gradient gel electrophoresis, and an average diameter of 7.95 ± 3.12 nm by dynamic light scattering (DLS, Figure 2d). To our delight, both homogenous bands in gel and narrow size distribution in DLS suggested no detectable aggregation of the assembled FA‐7CPT‐3WJ nanoparticles. This further demonstrated the advantage of RNA in solubilizing hydrophobic drugs and confirmed the accurate chemical entity of CPT‐RNA nanoparticles.

**Figure 2:**
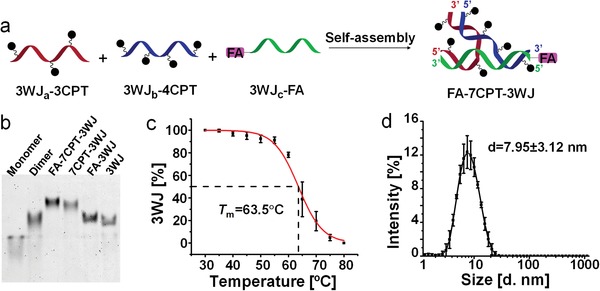
Assembly and characterization of FA‐7CPT‐3WJ nanoparticles. a) Illustration of FA‐7CPT‐3WJ assembly (triazole linkage is not shown, pink rectangle represents FA and black dots represent CPT prodrug). b) Stepwise assembly of FA‐7CPT‐3WJ with control 3WJs, evaluated by native PAGE. c) Melting curve of assembled FA‐7CPT‐3WJ in PBS buffer (*n* = 3, error bars are presented as mean ± SD). d) Size distribution of FA‐7CPT‐3WJ, measured by DLS (*n* = 3, error bars are presented as mean ± SD).

Ester bond has been proven to be enzymatically labile in the presence of endogenous esterase and was widely used as a cleavable linker in prodrug design.^24^ Our CPT prodrug molecules were conjugated to RNA oligos via ester bonds that allow the enzymatic release of the parent CPT in serum plasma and intracellular cytoplasm where esterase exists.^25^ To mimic the in vivo environment, 50% of fetal bovine serum was used to treat a single CPT‐displaying RNA (3WJ_a_‐CPT), and the resulting products were analyzed on a denaturing PAGE. The CPT release yield increased over incubation time and 50% of CPT release was observed at about 7.7 h (Figure S4, Supporting Information).

Folate receptor (FR) is frequently overexpressed on the tumor cell surface, and the dissociation constant between FR and its ligand, FA, is found to be in nanomolar range.^26^ For this reason, FA has been frequently used as a targeting ligand for the delivery of therapeutics.^27^ To evaluate the specific cell targeting of our RNA nanoparticles, Alexa647 dye has been displayed on all 3WJ samples. Fluorescent FA‐7CPT‐3WJ (FA‐7CPT‐3WJ‐Alexa647) and proper controls at different concentrations (25 × 10^−9^, 100 × 10^−9^, and 400 × 10^−9^ m) were incubated with KB cells for flow cytometry analysis. In the presence of FA ligand, the fluorescent FA‐7CPT‐3WJ nanoparticles showed strong binding to KB cells in a concentration‐dependent manner, compared to the one without FA (**Figure** **3**a). Interestingly, we found 7CPT‐3WJ exhibited higher binding affinity to cells compared to 3WJ without CPT at 100 × 10^−9^ m. The conjugation of hydrophobic CPT to 3WJ scaffold probably enhanced the nonspecific binding to the cell membrane. In contrast, FA‐7CPT‐3WJ showed a similar binding affinity as FA‐3WJ, which demonstrated FA dominated the binding process by FA–FR interaction with much higher affinity than nonspecific hydrophobic interaction caused by CPT (Figure 3a). To confirm the specific targeting effect caused by FA–FR recognition, HepG2 cells were used as a negative control as it was known not to overexpress folate receptor on cell surface.^28^ To our delight, FA‐7CPT‐3WJ‐Alexa647 showed little binding to HepG2 cells (Figure S5, Supporting Information). In addition, confocal microscope imaging confirmed the efficient binding and internalization of fluorescent RNA nanoparticles into KB cells. FA‐7CPT‐3WJ‐Alexa647 treated cells showed high intracellular fluorescence signal (Figure 3b). In contrast, low signal was detected for the control RNA nanoparticles without FA ligand. The specificity of FA–FR interaction was further confirmed by the addition of excess FA as a binding competitor during the incubation of RNA nanoparticles with KB cells. As a result, the internalization of FA‐7CPT‐3WJ‐Alexa647 nanoparticles to KB cells was significantly inhibited by the excess FA. These results together demonstrate the FA‐displaying RNA nanoparticles specifically bind to FR‐overexpressed tumor cells and are further internalized into the cells efficiently by receptor‐mediated endocytosis.

**Figure 3:**
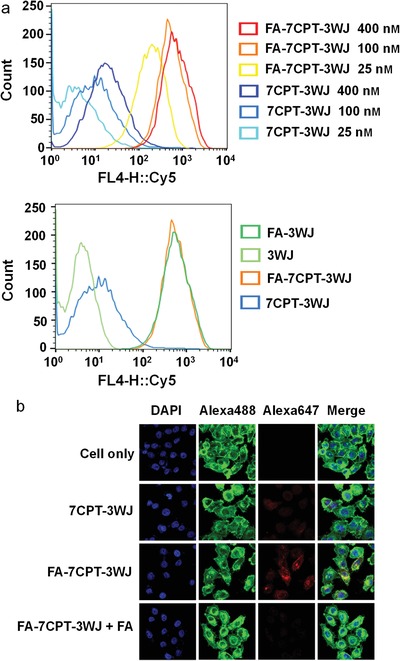
In vitro cellular binding and internalization of Alexa647‐RNA nanoparticles. a) Flow cytometry assay showing the RNA nanoparticles binding to KB cells (the concentration is based on assembled RNA nanoparticles, the concentration of each sample in the lower flow cytometry experiment is 100 × 10^−9^ m). b) Confocal microscope images indicating the internalization of RNA nanoparticles into KB cells (blue: nuclei; green: cytoskeleton; red: RNA nanoparticles; the concentration of each fluorescent RNA nanoparticles is 100 × 10^−9^ m and FA concentration is 100 × 10^−6^ m).

To determine the half maximal inhibitory concentration (IC50) of CPT‐RNA nanoparticles, dose‐dependent MTT (3‐(4,5‐dimethylthiazol‐2‐yl)‐2,5‐diphenyltetrazolium bromide) assay was performed at different time points, and inverted microscope was used to compare cell morphology after 48 h. The cell viability results demonstrated CPT‐RNA nanoparticles inhibited KB cells growth in a concentration‐dependent manner (**Figure** **4**a), while RNA nanoparticles without CPT did not show obvious inhibition (Figure S6, Supporting Information). Based on the growth inhibition results, IC50 for FA‐7CPT‐3WJ on KB cells was around 0.4 × 10^−6^ m (CPT concentration). However, IC50 for HepG2 cells was much larger (Figure S7, Supporting Information). This discrepancy on IC50 further supports the targeted delivery caused by FA–FR interaction. In addition, CPT‐RNA nanoparticles presented a delayed inhibition on tumor cell growth compared with CPT (IC50 was around 0.05 × 10^−6^ m). More specifically, when comparing FA‐7CPT‐3WJ at 72 h with CPT at 48 h, samples of higher concentrations have comparable performance. This was also supported by the comparison of FA‐7CPT‐3WJ at 48 h with CPT at 24 h. This delayed action may be caused by the slow release of CPT from RNA nanoparticles. Different mechanisms of cell internalization for FA‐7CPT‐3WJ and CPT could also lead to this delayed inhibition. Furthermore, inverted microscope images showed that both FA‐7CPT‐3WJ‐ and 7CPT‐3WJ‐treated cells exhibited poor cell growth after 48 h incubation, as characterized by enlarged nuclei and elliptical membranes, as well as the reduced cell density comparable to those of CPT and CPT prodrug treatment groups (Figure S8, Supporting Information). In contrast, FA‐3WJ‐ and 3WJ‐treated cells exhibited healthy cell growth, indicating the safety of using RNA nanoparticles as drug carriers. These results demonstrated that parent CPT drug was released from the RNA nanoparticles and retained its pharmacological activity to inhibit tumor cell growth.

**Figure 4:**
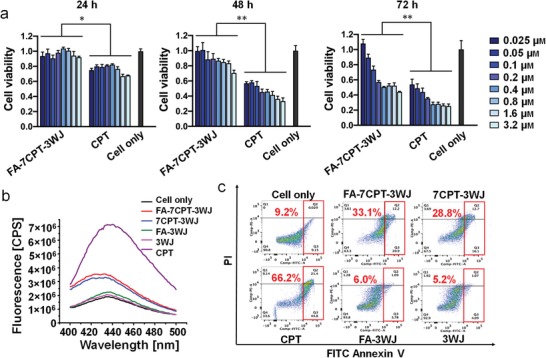
In vitro dose‐dependent tumor cell growth inhibition and apoptosis effects of CPT‐RNA nanoparticles. a) MTT assay showing cell viability at 24, 48, and 72 h post treatments (*n* = 3, results are presented as mean ± SD, *p < 0.05, **p < 0.01; concentration is based on CPT). b) Caspase‐3 assay indicating cellular apoptotic effects 48 h post treatments. c) Apoptotic effects assayed by PI/Annexin V‐FITC dual staining and fluorescence‐activated cell sorting analysis.

Caspase‐3 as an early cell apoptosis marker was also monitored during the treatments. Elevation of caspase‐3 is reflected by the increased fluorescence signal from the caspase‐3 fluorogenic substrate. Consistent with the MTT assay, caspase‐3 measurement confirmed tumor cell apoptosis induced by CPT‐RNA nanoparticles (Figure 4b). This caspase‐3 induction was time sensitive (Figure S9, Supporting Information). Propidium iodide (PI) and FITC Annexin V double staining analysis also confirmed the apoptosis induced by CPT‐RNA nanoparticles. The results showed 33.1% and 28.8% of cells underwent apoptosis after being treated with FA‐7CPT‐3WJ and 7CPT‐3WJ for 48 h, respectively, whereas FA‐3WJ and 3WJ did not induce obvious apoptosis (Figure 4c). As a positive control, CPT‐treated cells induced 66.2% apoptosis. The results are consistent with caspase‐3 assay, and further confirm that the cell viability change was due to cell apoptosis triggered by the released CPT.

Given the demonstration of specific tumor cell binding and apoptosis effect, we set out to study in vivo tumor suppression in human tumor xenograft model. After the tumors grew to approximately 50 mm^3^, mice bearing KB xenograft were randomly divided into four groups of PBS, CPT, 7CPT‐3WJ, and FA‐7CPT‐3WJ (*n* = 5) and administered via IV injection at the dose of 4.3 mg kg^−1^ (CPT/body weight, 2.47 × 10^−3^ m in 0.1 mL) on day 1, 3, 5, and 7 for a total of four injections. CPT dissolved very poorly in aqueous solution and many previous studies used intraperitoneal injection or intramuscular injection with special formulations.[qv: 17,20b,c] To ensure comparable administrations, free CPT drug was formulated in a reported method (10% DMSO and 5% Tween 80)^29^ and studied by IV injection. Among all treatment groups, FA‐7CPT‐3WJ nanoparticles exhibited the most significant tumor suppression based on tumor size (**Figure** **5**a). CPT and 7CPT‐3WJ showed similar tumor suppression; however, severe acute toxicity was observed for the free CPT group, and over 50% fatalities were found by day 9 during the experiments. Other studies also reported emergent toxicity with different administration methods for free CPT drug.[qv: 20b,c] RNA nanoparticles without CPT loading were not included in this in vivo experiment, as it has been previously reported to display minimal toxicity by histological analysis in mice.[qv: 15a,b] In contrast, both CPT‐RNA nanoparticles did not cause any fatality or obvious weight changes during experiments (Figure 5b), suggestive of a favorable safety profile of the CPT‐RNA nanoparticles. Due to the recognition between FA and its overexpressed receptor on KB cells, FA‐7CPT‐3WJ outperformed 7CPT‐3WJ in tumor suppression by about 20% more reduction in tumor weight (Figure 5c). These results demonstrated CPT‐RNA nanoparticles with targeting ligand (FA‐7CPT‐3WJ) were the most efficacious group with significantly reduced toxicity and enhanced tumor inhibition efficiency compared to the free drug formulation.

**Figure 5:**
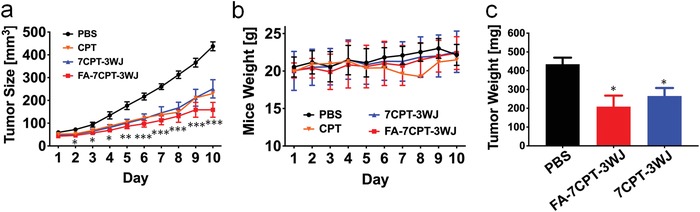
Tumor suppression of CPT‐RNA nanoparticles using KB tumor xenograft. a) Tumor growth curve (*p < 0.05, **p < 0.01, ***p < 0.001, error bars indicate standard error of the mean (SEM)). b) Mice weight curve (error bars indicate SD). c) Tumor weight measurement after harvested at Day 10 (*p < 0.05, error bars indicate SEM).

In summary, RNA nanoparticles were used for solubilizing hydrophobic antitumor drug and for targeted delivery in a human tumor xenograft model. High loading of the poorly soluble CPT to the hydrophilic RNA nanoparticles dramatically improved drug solubility in physiological conditions while maintaining therapeutic effect. The resulting therapeutic RNA nanoparticles had the advantages of well‐defined structure, precise drug loading, high thermal and chemical stability, and targeted delivery. The potent CPT was enzymatically released from the RNA vehicles by esterase in vivo, triggering tumor cell apoptosis and inhibiting tumor growth effectively. The in vitro and in vivo data collectively demonstrated the feasibility of RNA nanoparticles for the safe and efficacious targeted delivery of hydrophobic antitumor drugs.

## Experimental Section

Detailed experimental procedures are summarized in the Supporting Information. All protocols involving animals were approved and performed under the supervision of The Ohio State University Institutional Animal Care and Use Committee (IACUC).

## Conflict of Interest

P.G. is the consultant of Oxford Nanopore Technologies and Nanobio Delivery Pharmaceutical Co. Ltd; he is the cofounder of Shenzhen P&Z Bio‐medical Co. Ltd and its subsidiary US P&Z Biological Technology LLC, as well as ExonanoRNA, LLC and its subsidiary Weina Biomedical LLC in Foshan.

## Supporting information

Supplementary

## References

[1] a) E. Bilensoy , Expert Opin. Drug Delivery 2010, 7, 795; 10.1517/17425247.2010.485983 20446858

[2] V. T. DeVita Jr. , E. Chu , Cancer Res. 2008, 68, 8643. 18974103 10.1158/0008-5472.CAN-07-6611

[3] H. Unal , N. Ozturk , E. Bilensoy , Beilstein J. Org. Chem. 2015, 11, 204. 25815071 10.3762/bjoc.11.22 PMC4362320

[4] M. Narvekar , H. Y. Xue , J. Y. Eoh , H. L. Wong , Aaps Pharmscitech 2014, 15, 822. 24687241 10.1208/s12249-014-0107-x PMC4113620

[5] R. Savla , J. Browne , V. Plassat , K. M. Wasan , E. K. Wasan , Drug Dev. Ind. Pharm. 2017, 43, 1743. 28673096 10.1080/03639045.2017.1342654

[6] a) A. Sparreboom , Z. L. van , E. Brouwer , W. J. Loos , B. P. de , H. Gelderblom , M. Pillay , K. Nooter , G. Stoter , J. Verweij , Cancer Res. 1999, 59, 1454; 10197613

[7] a) T. M. Allen , P. R. Cullis , Adv. Drug Delivery Rev. 2013, 65, 36; 10.1016/j.addr.2012.09.037 23036225

[8] a) K. S. Soppimath , T. M. Aminabhavi , A. R. Kulkarni , W. E. Rudzinski , J. Controlled Release 2001, 70, 1; 10.1016/s0168-3659(00)00339-4 11166403

[9] a) O. Veiseh , J. W. Gunn , M. Zhang , Adv. Drug Delivery Rev. 2010, 62, 284; 10.1016/j.addr.2009.11.002 PMC2827645 19909778

[10] W. H. De Jong , P. J. Borm , Int. J. Nanomed. 2008, 3, 133. 10.2147/ijn.s596 PMC2527668 18686775

[11] a) D. Shu , Y. Shu , F. Haque , S. Abdelmawla , P. Guo , Nat. Nanotechnol. 2011, 6, 658; 21909084 10.1038/nnano.2011.105 PMC3189281

[12] a) D. W. Binzel , E. F. Khisamutdinov , P. Guo , Biochemistry 2014, 53, 2221; 24694349 10.1021/bi4017022 PMC4004221

[13] a) X. Piao , H. Wang , D. W. Binzel , P. Guo , RNA 2018, 24, 67; 29051199 10.1261/rna.063057.117 PMC5733572

[14] D. Binzel , Y. Shu , H. Li , M. Sun , Q. Zhang , D. Shu , B. Guo , P. Guo , Mol. Ther. 2016, 24, 1267. 27125502 10.1038/mt.2016.85 PMC5088763

[15] a) Y. Zhang , M. Leonard , Y. Shu , Y. Yang , D. Shu , P. Guo , X. Zhang , ACS Nano 2017, 11, 335; 27966906 10.1021/acsnano.6b05910 PMC5488869

[16] a) F. Pi , H. Zhang , H. Li , V. Thiviyanathan , D. G. Gorenstein , A. K. Sood , P. Guo , Nanomedicine 2016, 13, 1183; 27890659 10.1016/j.nano.2016.11.015 PMC5426907

[17] B. C. Giovanella , H. R. Hinz , A. J. Kozielski , J. S. Stehlin Jr. , R. Silber , M. Potmesil , Cancer Res. 1991, 51, 3052. 2032244

[18] V. J. Venditto , E. E. Simanek , Mol. Pharmaceutics 2010, 7, 307. 10.1021/mp900243b PMC3733266 20108971

[19] a) H. G. Lerchen , J. Baumgarten , B. K. von dem , T. E. Lehmann , M. Sperzel , G. Kempka , H. H. Fiebig , J. Med. Chem. 2001, 44, 4186; 11708920 10.1021/jm010893l

[20] a) R. Omar , Y. L. Bardoogo , E. Corem‐Salkmon , B. Mizrahi , J. Controlled Release 2017, 257, 76; 10.1016/j.jconrel.2016.09.025 27677603

[21] W. A. Henne , S. A. Kularatne , W. Yala‐Lopez , D. D. Doorneweerd , T. W. Stinnette , Y. Lu , P. S. Low , Bioorg. Med. Chem. Lett. 2012, 22, 709. 22100311 10.1016/j.bmcl.2011.10.042

[22] a) G. Thiagarajan , A. Ray , A. Malugin , H. Ghandehari , Pharm. Res. 2010, 27, 2307; 20552256 10.1007/s11095-010-0179-6 PMC3092430

[23] A. M. Saetern , G. E. Flaten , M. Brandl , Aaps Pharmscitech 2004, 5, e40. 15760073 10.1208/pt050340 PMC2750263

[24] a) B. M. Liederer , R. T. Borchardt , J. Pharm. Sci. 2006, 95, 1177; 16639719 10.1002/jps.20542

[25] a) L. Tian , Y. Yang , L. M. Wysocki , A. C. Arnold , A. Hu , B. Ravichandran , S. M. Sternson , L. L. Looger , L. D. Lavis , Proc. Natl. Acad. Sci. U. S. A. 2012, 109, 4756; 22411832 10.1073/pnas.1111943109 PMC3323987

[26] a) B. A. Kamen , A. Capdevila , Proc. Natl. Acad. Sci. U. S. A. 1986, 83, 5983; 3461471 10.1073/pnas.83.16.5983 PMC386421

[27] P. S. Low , A. C. Antony , Adv. Drug Delivery Rev. 2004, 56, 1055. 10.1016/j.addr.2004.02.003 15094205

[28] D. Hu , Z. Sheng , S. Fang , Y. Wang , D. Gao , P. Zhang , P. Gong , Y. Ma , L. Cai , Theranostics 2014, 4, 142. 24465272 10.7150/thno.7266 PMC3900799

[29] D. J. Jang , C. Moon , E. Oh , Biomed. Pharmacother. 2016, 80, 162. 27133053 10.1016/j.biopha.2016.03.018

