# Supplementary material for: RNA Nanotechnology to Solubilize Hydrophobic Antitumor Drug for Targeted Delivery
Source: Adv Sci (Weinh). 2019 Sep 30;6(22):1900951. doi: 10.1002/advs.201900951 (PMC6864502; doi:10.1002/advs.201900951)
Supplement: Supplementary file 1 — Supplementary [file ADVS-6-1900951-s001.pdf]

Copyright WILEY-VCH Verlag GmbH & Co. KGaA, 69469 Weinheim, Germany, 2019.

ADVANCED SCIENCE
Open Access

Supporting Information

for *Adv. Sci.,* DOI: 10.1002/advs.201900951

RNA Nanotechnology to Solubilize Hydrophobic Antitumor Drug for Targeted Delivery

*Xijun Piao, Hongran Yin, Sijin Guo, Hongzhi Wang, and Peixuan Guo**

Copyright WILEY-VCH Verlag GmbH & Co. KGaA, 69469 Weinheim, Germany, 2019.

Supporting Information

RNA Nanotechnology to Solubilize Hydrophobic Antitumor Drug for Targeted Delivery

*Xijun Piao, Hongran Yin, Sijin Guo, Hongzhi Wang, and Peixuan Guo**

Materials and Equipments

(*S*)-(+)-Camptothecin, 4-Dimethylaminopyridine (DMAP), Copper(I) Bromide (CuBr), Tris[(1-benzyl-1*H*-1,2,3-triazol-4-yl)methyl]amine (TBTA) were purchased from Sigma-Aldrich (St. Louis, MO). 1-Ethyl-3-(3-dimethylaminopropyl)carbodiimide hydrochloride (EDC) was obtained from Thermo Fisher Scientific (Rockford, IL). 6-Azidohexonic acid was ordered from Chem-Impex International, Inc (Wood Dale, IL). All solvents for organic synthesis were supplied by Sigma-Aldrich (St. Louis, MO). Chemicals, solvents and other supplies for solid-phase oligonucleotide synthesis and HPLC were purchased from Bioautomation (Irving, TX) and Glen Research (Sterling, VA). Folate(5')-displaying $3WJ_c$ oligonucleotide ($3WJ_c$-FA) was supplied by NanoBio Delivery Pharmaceutical Co., Ltd (Columbus, OH). FA-displaying phosphoramidite was used at the last position (5') in the automated synthesis of $3WJ_c$-FA and no post-synthesis conjugation was required. Folate(5')-Alexa647(3') $3WJ_c$ oligonucleotide was supplied by TriLink Biotechnologies (San Diego, CA). Chemicals for gel electrophoresis were purchased from Fisher Scientific (Fair Lawn, NJ) and Bio-Rad (Hercules, CA). Mediums and buffers for cell culture were supplied by Thermo Scientific and Sigma-Aldrich. 4-5 weeks female athymic nu/nu mice were purchased from Taconic (Rensselaer, NY).

NMR was done on Bruker AV300. Solid-phase oligonucleotide synthesis was performed on Biosset ASM-800 synthesizer. HPLC was performed on Agilent 1260 Infinity

with Agilent PLRP-S 8μM 300Å column. MALDI-TOF mass spectrometry was carried out on Bruker MicroFlex. UV/Vis absorbance was measured on NanoDrop 2000 by Thermo Scientific. Gel electrophoresis system was from Bio-Rad, and all gels were scanned on GE Typhoon FLA 7000. The temperature gradient gel electrophoresis (TGGE) system was from Biometra. Dynamic light scattering (DLS) was done on Malvern zetasizer. Confocal microscopy was done on the Olympus FV 3000 Spectra Confocal system and flow cytometry was done on Becton Dickinson FACSCalibur Flow Cytometer in OSUCCC Analytical Cytometry Shared Resource (ACSR). Olympus IX71 Inverted microscope was used for cell morphology observation.

Synthesis of CPT prodrug

CPT prodrug synthesis followed a previous publication.[1] Briefly, CPT (78 mg, 0.22 mmol), EDC (61 mg, 0.32 mmol), DMAP (40 mg, 0.32 mmol), and 6-Azidohexonic acid (51 mg, 0.32 mmol) were dissolved in 10 mL dichloromethane and stirred at room temperature. The reaction was monitored by TLC to ensure the complete consumption of CPT. After the reaction was completed, it was washed with 1 M HCl three times and the organic layer was dried over anhydrous $MgSO_4$. Upon the removal of dichloromethane by rotavaporation, the crude product was purified by recrystallization in methanol: dichloromethane (95: 5) followed by washing the product with a small amount of cold methanol. The synthesis yield was 65%. ^{1}H NMR ($CDCl_3$, 300 MHz): δ (ppm) 8.40 (s, 1H), 8.22 (d, *J*=8.7Hz,1H), 7.94 (d, *J*=8.1Hz, 1H), 7.84 (t, *J*=6.9Hz, 1H), 7.68 (t, *J*=6.9Hz, 1H), 7.20 (s, 1H), 5.68 (d, *J*=17.1Hz, 1H), 5.41 (d, *J*=17.4Hz, 1H), 3.23 (t, *J*=7.1Hz, 2H), 2.52 (m, 2H), 2.28 (m, 1H), 2.16 (m, 1H), 1.67 (m, 2H), 1.59 (m, 2H), 1.46 (m, 2H), 0.98 (t, *J*=7.5Hz, 3H)

Synthesis of chemically-modified RNA oligos

Standard procedures were followed for solid-phase oligonucleotide synthesis at 1 μmol scale. All pyrimidines were chemically modified by 2'-fluoro (2'-F) or 2'-O-propargyl and all purine were 2'-OH. The sequences are listed, and underlined letters indicate the position of 2'-O-propargyl nucleotides if they are incorporated in the sequences.

3WJ$_a$ (18-nt): 5'-UUGCCAUGUGUAUGUGGG-3'

3WJ$_b$ (20-nt): 5'-CCCACAUACUUUGUUGAUCC-3'

3WJ$_c$ (16-nt): 5'-GGAUCAAUCAUGGCAA-3'

Conjugation of CPT prodrug to alkyne-displaying RNA oligos

CPT prodrug was conjugated to alkyne-displaying RNA oligos via Copper-catalyzed azide-alkyne cycloaddition (CuAAC). 100 mM CuBr solution (tBuOH: DMSO= 1:3) was mixed with 100 mM TBTA (tBuOH: DMSO= 1:3) in a ratio of 1:2 to yield Cu-TBTA complex. 10 µL of such freshly prepared Cu-TBTA solution was added to 60 µL CPT prodrug solution (DMSO, 20 mM) followed by adding 50 µL alkyne-displaying RNA oligos (3WJ$_a$-3alkyne or 3WJ$_b$-4alkyne) solution (water, 1-2.5 mM measured by RNA absorbance at 260 nm). Additional 130 µL DMSO was added to keep CPT prodrug soluble. The reaction was done overnight at room temperature. Water was added to reach 400 µL (total volume) followed by adding 40 µL 3 M sodium acetate (pH 5.2) and 1100 µL ethanol (200 proof). The tube was stored at -20 ºC for at least two hours to precipitate CPT-RNA conjugate. After centrifugation at maximal speed (13100 RPM) at 4 ºC for 30 minutes and washing the pellet with 70% cold ethanol, the pellet was dried by spin vacuum. 100 µL water was used to dissolve CPT-RNA conjugate and the partially precipitated CPT prodrug was removed by filtration. CPT-RNA conjugate was further purified by reverse phase HPLC (Solvent A: 0.1 M TEAA (triethylamine acetate) in water and solvent B: 75% acetonitrile and 25% water with 0.1 M TEAA).

Confirmation of CPT-RNA conjugation

Successful CPT-RNA conjugation was resolved on 16% 8 M urea denaturing PAGE or reverse-phase HPLC with an increasing gradient of solvent B (75% Acetonitrile with 0.1 M TEAA) from 5% to 90 % in one hour. Maldi-Tof Mass Spectrometry was performed on Bruker MicroFlex using 3-hydroxypicolinic acid (3-HPA) as the matrix.

Evaluation of improved water solubility by CPT-RNA conjugates

The molarity of CPT-RNA conjugate was measured according to the absorbance at 260 nm which was mainly resulted from RNA. For $3WJ_a$-3CPT, the concentration of CPT was readily obtained by multiplying CPT-RNA molarity by three. For CPT free drug, the calculated amount of CPT was weighed on analytical balance and mixed with expected amount of water or DMSO. For CPT free drug in water, a homogeneous suspension was made by sonication for 5 minutes and UV absorbance of these saturated aqueous solutions were measured after the high-speed centrifugation to remove undissolved CPT free drug. Absorbance at 354 nm was recorded for all three solutions and plotted against CPT molarity (theoretical molarity was used for CPT free drug in water as its solubility in water was poor).

Release of CPT in serum

10 µM CPT-RNA conjugate ($3WJ_a$-CPT) was incubated in 50% FBS. 3 µL samples were taken at different time points (up to 12 hours) and fast frozen on dry ice and stored at -80 ºC until analyzed on 16% 8 M urea denaturing PAGE. CPT release yield against incubation time was then quantified by ImageJ.

Assembly of RNA 3WJ samples

All RNA 3WJ samples were prepared by mixing three separate strands ($3WJ_a$, $3WJ_b$, and $3WJ_c$) at equimolar concentration in PBS buffer, annealed at 85 ºC for 5 minutes, and

allowed to cool to room temperature over 40 minutes. The assembly was characterized on 12% native PAGE using TBE buffer (120V, 4 ºC, 90 minutes). All assembled RNA 3WJ samples studied in cell and animal experiments were sterilized by 0.22 µm filter. Filtered 3WJ samples were also used in the measurements of dynamic light scattering.

T_m measurement of RNA 3WJ samples

T_m of assembled RNA 3WJ samples were measured on TGGE. Assembled RNA 3WJ samples (5 µM, 2 µL) were loaded into each lane of a 12% TBE native PAGE and ran at 100 V for 10 minutes. After all sample entered the gel, it was transferred to TGGE where the lanes were heated at increasing temperatures gradient from 30 ºC to 80 ºC across the gel while running at 100 V for 1 hour. The T_m was determined when 50% of the assembled RNA 3WJ sample dissociated.

Cell culture

Human KB cells (American Type Culture Collection, ATCC) cultured in RPMI-1640-folate deficient (Thermo Scientific) medium containing 10% FBS and 1% Penicillin-Streptomycin in a 37 ºC incubator under 5% CO_2 and a humidified atmosphere.

In vitro cell binding and internalization assay

Concentration is based on assembled RNA nanoparticles. For flow cytometry assay, 25 nM, 100 nM and 400 nM Alexa647 labeled FA-7CPT-3WJ and 7CPT-3WJ nanoparticles were incubated with KB cells at 37 ºC for 1 hour. Besides, 100 nM Alexa647 labeled FA-3WJ and 3WJ without CPT were also used for incubation as control groups. 100 nM Alexa647 labeled FA-7CPT-3WJ and 7CPT-3WJ nanoparticles were also incubated with HepG2 cells at 37 ºC for 1 hour. After washing with PBS twice, the cells were resuspended in PBS for flow

cytometry analysis. It was performed by OSUCCC Analytical Cytometry Shared Resource (ACSR). The data was analyzed by FlowJo software.

For confocal microscope imaging, KB cells were seeded on glass slides at 70% confluence one day before treatment. 100 nM FA-7CPT-3WJ-Alexa647 and 7CPT-3WJ-Alexa647 were incubated with cells at 37 ºC for 1 hour. For specificity study, 100 µM folic acid were added to FA-7CPT-3WJ-Alexa647 for co-incubation with cells. After incubation, the cells were washed with PBS and fixed by 4% paraformaldehyde (PFA). The cytoskeleton of cells was stained by Alexa Fluor 488 Phalloidin after pre-treated by 0.1% Triton-X 100 for 5 minutes. The slides with cells were finally mounted with DAPI for nucleus staining. The internalization assay was analyzed by Olympus FV-3000 Spectra Confocal Microscope.

***In vitro* cytotoxicity effects study**

All concentrations are calculated based on CPT. 5×10^3 KB cells or HepG2 cells were seeded in 96-well plates one day before treatment. 0.025 µM, 0.05 µM, 0.1 µM, 0.2 µM, 0.4 µM, 0.8 µM, 1.6 µM, 3.2 µM of FA-7CPT-3WJ and CPT were added to the wells in triplets. In a separate experiment, 0.2 µM, 0.8 µM, and 3.2 µM of FA-7CPT-3WJ, 7CPT-3WJ, FA-3WJ and 3WJ nanoparticles were added to the wells of KB cells in triplets. The same concentration of CPT and CPT-prodrug alone were also used as controls for the assay. The plates were incubated at 37 ºC in a humidified, 5% CO_2 atmosphere. After 48-hour incubation, the cells of each treatment group were observed by Inverted Microscope to compare their morphology difference. At 24h, 48h, and 72h, MTT assay was conducted by CellTiter 96 Non-Radioactive Cell Proliferation Assay (Promega) following manufacture's instruction. 15 µL of the MTT Dye Solution was added to each well for incubation at 37 ºC for 4 hours. Then, 100 µL of Solubilization Solution/ Stop Mix was added to each well to incubate for 2 additional hours. The plate was gently shaken by shaker to get a uniformly colored solution. The absorbance at 570 nm was read by Synergy 4 microplate reader (Bio-Tek).

In vitro apoptosis study

For Caspase-3 assay, KB cells were seeded to 24-well plate overnight. The cells were treated with FA-7CPT-3WJ, 7CPT-3WJ, FA-3WJ, 3WJ as well as CPT alone as a control. The Caspase-3 activity was measured by Caspase-3 Assay Kit (BD Pharmingen) following the manufacture's instruction. Briefly, cell lysate was collected after 6h, 24h and 48h treatment using Cell Lysis Buffer in the kit. For each group, 40 µL of cell lysate was incubated with 2 µL reconstituted Ac-DEVD-AMC substrate in 80 µL of 1× HEPES buffer and incubated at 37 ºC for 1 hour. The AMC released from Ac-DEVD-AMC was measured by a Fluorolog spectroflurometer (Horiba Jobin Yvon) with excitation wavelength of 380 nm over an emission wavelength of 400-500 nm.

For PI& FITC Annexin V staining assay, KB cells were seeded into 24-well plate overnight. The cells were treated with samples described above. After 48-hour incubation, the assay was conducted using FITC Annexin V Apoptosis Detection Kit I (BD Pharmingen). The cells were trypsinized, washed by PBS twice and re-suspended in the 1× Binding Buffer (1×10^6 cells/mL). 100 µL of solution was transferred to flow tube. 5 µL FITC Annexin V and 5 µL propidium iodide (PI) were added for 15-minute incubation at room temperature. Finally, 400 µL 1× Binding Buffer was added to each tube for analysis by flow cytometry within 1 hour.

Animal trials

All protocols involving animals were performed under the supervision of The Ohio State University Institutional Animal Care and Use Committee (IACUC). To generate xenograft model, 4-5 weeks female athymic nu/nu mice were purchased from Taconic. The mice were fed with folate deficient diet for 2 weeks before building the tumor model. 2.5×10^6 KB cells resuspended in 100 µL DPBS were injected to the shoulder of nude mice

subcutaneously. The treatment started after the tumor size reached about 50 mm^3 for tumor regression study.

When the tumor size reached about 50 mm^3, KB tumor xenograft bearing mice were randomly divided into 4 groups (n=5). PBS, FA-7CPT-3WJ, 7CPT-3WJ and CPT alone (Formulated in 10% DMSO and 5% Tween 80) were injected to the mice for a total of four doses at 4.3 mg/kg (CPT/mice weight) every other day. PBS treated mice were served as a control group. The tumor volume was measured every day and calculated as (length × width^2)/2. The mice weight was also recorded every day to monitor whether the treatment caused toxicity and weight lost. At day 10 post-treatment, the mice were sacrificed, and tumors were harvested. The tumor weight of each mice was measure and recorded.

Additional Results

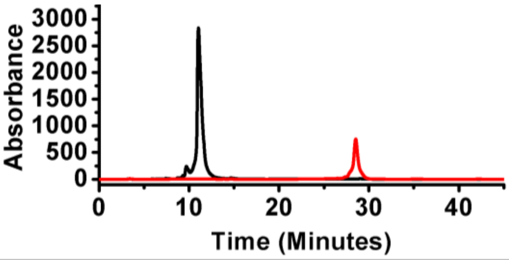

Figure S1. Comparison of reverse-phase HPLC spectra (Absorbance at 260nm) of 3WJ$_b$-4alkyne (Black) and 3WJ$_b$-4CPT after purification (Red).

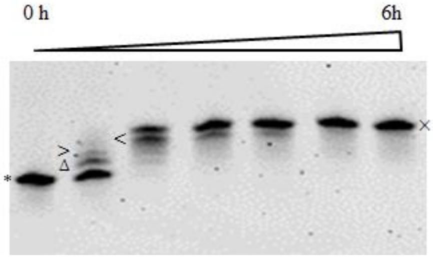

Figure S2. 3WJ$_b$-4CPT conjugation with increasing reaction time up to 6 h, evaluated by denaturing PAGE (* indicates 3WJ$_b$-4alkyne; $^{\Delta}$ indicates 3WJ$_b$-1CPT; $^{>}$ indicates 3WJ$_b$-2CPT; $^{<}$ indicates 3WJ$_b$-3CPT; $^{\times}$indicates 3WJ$_b$-4CPT).

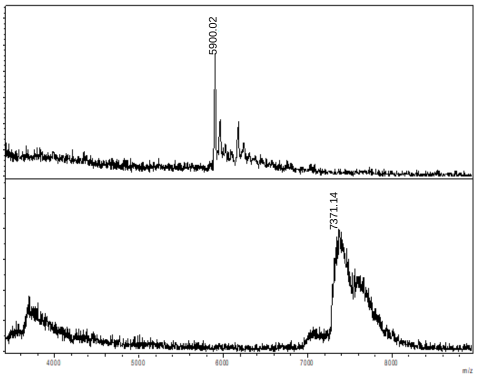

Figure S3. Maldi-Tof Mass Spectra for 3WJ$_a$-3alkyne (Top) and 3WJ$_a$-3CPT (Bottom).

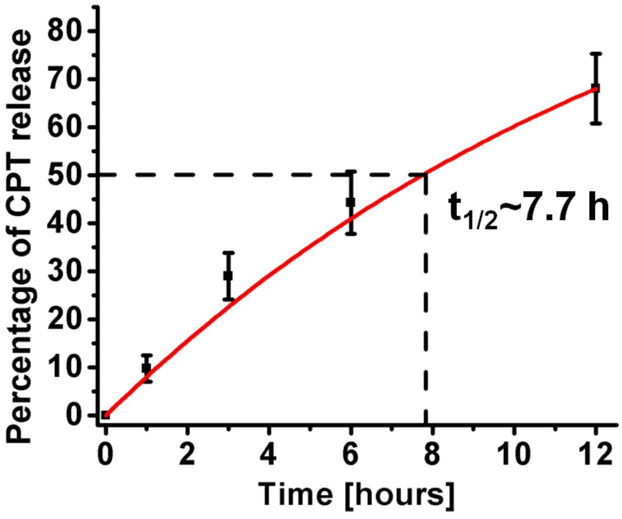

Figure S4. CPT release from CPT-RNA conjugate (3WJ$_a$-CPT) in 50% FBS over time (n=3, results are presented as mean ± SD).

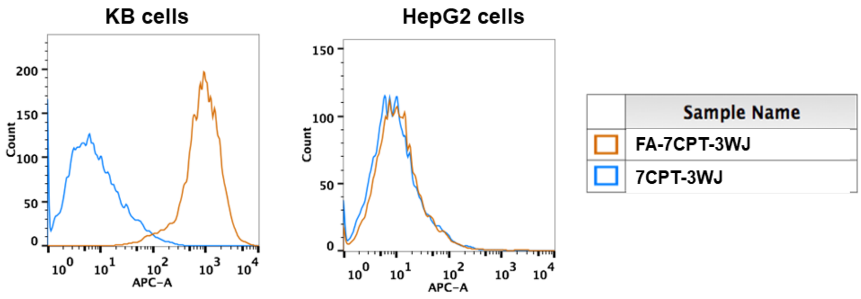

Figure S5. Flow cytometry assay showing the RNA nanoparticles binding to KB and HepG2 cells (100 nM of RNA-CPT nanoparticles and the concentration is based on assembled RNA nanoparticles).

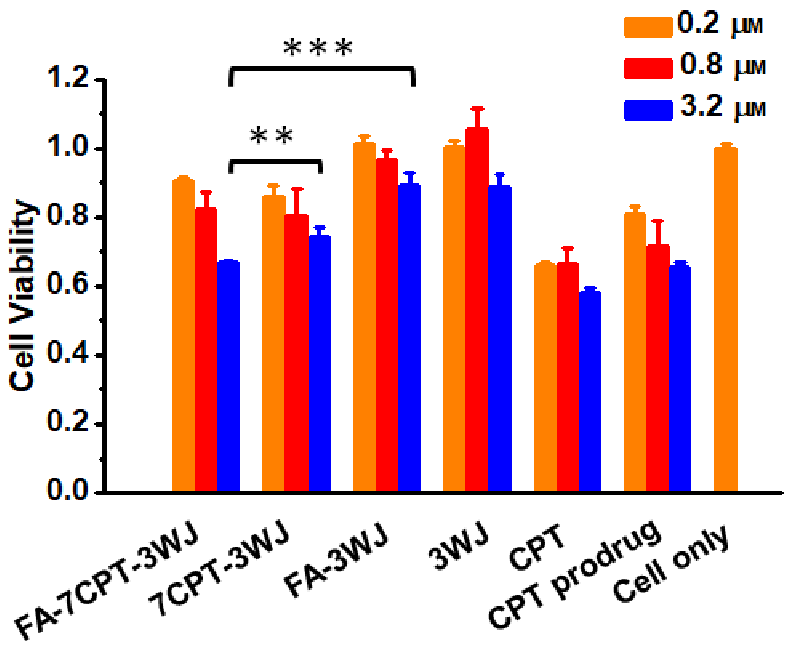

Figure S6. MTT assay showing cell viability 72-h post treatments (n=3, results are presented as mean ± SD, **p<0.01, ***p<0.001. Concentration is based on CPT, sample 3WJ has the same amount of RNA as FA-7CPT-3WJ and 7CPT-3WJ).

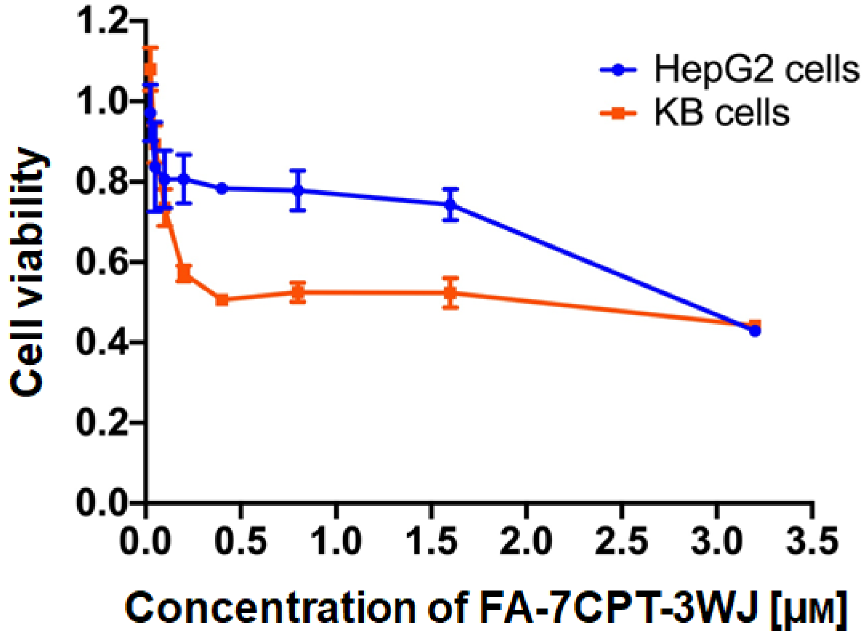

Figure S7. Comparison of dose-dependent FA-7CPT-3WJ cytotoxicity at 72h on KB and HepG2 cells

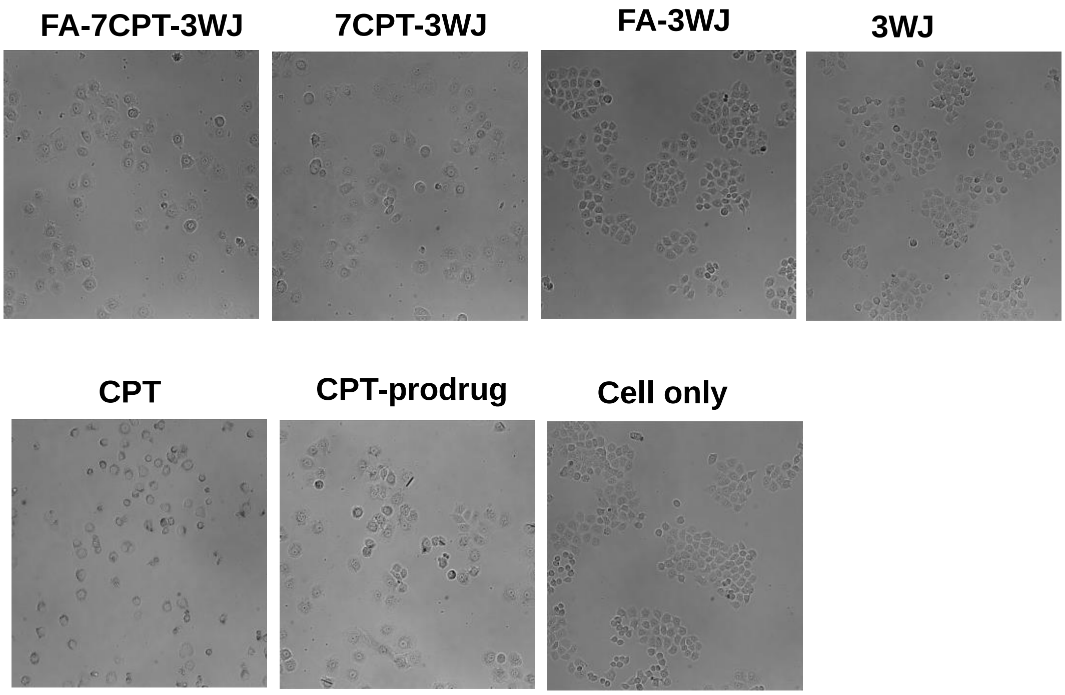

Figure S8. Microscope images showing cytotoxicity CPT-RNA conjugate after 48h incubation with KB cells.

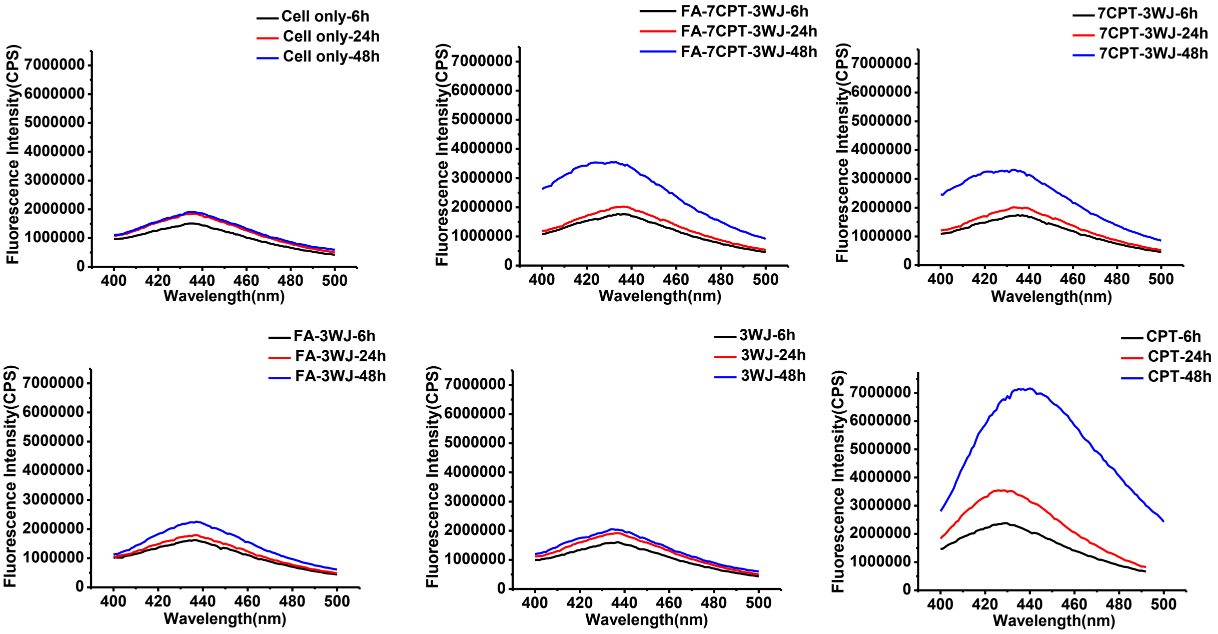

Figure S9. Time course apoptosis induction by Caspase-3 assay after treatments.

Reference

[1] O. Y. Zolotarskaya, A. F. Wagner, J. M. Beckta, K. Valerie, K. J. Wynne, H. Yang, *Mol. Pharm.* **2012**, *9*, 3403.
